# Examination of betahistine bioavailability in combination with the monoamine oxidase B inhibitor, selegiline, in humans—a non-randomized, single-sequence, two-period titration, open label single-center phase 1 study (PK-BeST)

**DOI:** 10.3389/fneur.2023.1271640

**Published:** 2023-10-18

**Authors:** Michael Strupp, Grant C. Churchill, Ivonne Naumann, Ulrich Mansmann, Amani Al Tawil, Anastasia Golentsova, Nicolina Goldschagg

**Affiliations:** ^1^Department of Neurology and German Center for Vertigo and Balance Disorders, LMU University Hospital, LMU Munich, Munich, Germany; ^2^Department of Pharmacology, University of Oxford, Oxford, United Kingdom; ^3^Department of Medical Information Sciences, Biometry and Epidemiology, Ludwig Maximilians University, Munich, Germany

**Keywords:** betahistine, selegiline, MAO-B inhibitor, phase 1 trial, Menière, vertigo

## Abstract

**Background:**

Betahistine was registered in Europe in the 1970s and approved in more than 80 countries as a first-line treatment for Menière's disease. It has been administered to more than 150 million patients. However, according to a Cochrane systematic review of betahistine and recent meta-analyses, there is insufficient evidence to say whether betahistine has any effect in the currently approved dosages of up to 48 mg/d. A combination with the monoamine oxidase B (MAO-B) inhibitor, selegiline, may increase the bioavailability of betahistine to levels similar to the well-established combination of L-DOPA with carbidopa or benserazide in the treatment of Parkinson's disease. We investigated the effect of selegiline on betahistine pharmacokinetics and the safety of the combination in humans.

**Methods:**

In an investigator-initiated prospective, non-randomized, single-sequence, two-period titration, open label single-center phase 1 study, 15 healthy volunteers received three single oral dosages of betahistine (24, 48, and 96 mg in this sequence with at least 2 days' washout period) without and with selegiline (5 mg/d with a loading period of 7 days). Betahistine serum concentrations were measured over a period of 240 min at eight time points (area under the curve, AUC0-240 min). This trial is registered with EudraCT (2019-002610-39) and ClinicalTrials.gov.

**Findings:**

In all three single betahistine dosages, selegiline increased the betahistine bioavailability about 80- to 100-fold. For instance, the mean (±SD) of the area under curve for betahistine 48 mg alone was 0.64 (+/-0.47) h^*^ng/mL and for betahistine plus selegiline 53.28 (+/-37.49) h^*^ng/mL. The half-life time of around 30 min was largely unaffected, except for the 24 mg betahistine dosage. In total, 14 mild adverse events were documented.

**Interpretation:**

This phase 1 trial shows that the MAO-B inhibitor selegiline increases betahistine bioavailability by a factor of about 80 to 100. No safety concerns were detected. Whether the increased bioavailability has an impact on the preventive treatment of Menière's disease, acute vestibular syndrome, or post-BPPV residual dizziness has to be evaluated in placebo-controlled trials.

**Clinical trial registration:**

https://clinicaltrials.gov/study/NCT05938517?intr=betahistine%20and%20selegiline&rank=1, identifier: NCT05938517.

## 1. Introduction

Betahistine is a structural analog of histamine with weak histamine H1 receptor agonist and more potent histamine H3 receptor antagonist properties. Betahistine has been used for various indications, namely for the prophylactic treatment of Menière's disease for over 50 years and for the symptomatic treatment of vertigo. It is approved for Menière's disease in more than 80 countries and has been used in over 130 million patients with a favorable safety profile (1). A randomized, placebo-controlled, multi-center trial (conducted by our institution), however, demonstrated that its approved dosage (48 mg/day), and even 144 mg/day, were not superior to placebo (2); this finding is supported by a systematic review (3) and Cochrane analysis (4). Nevertheless, a survey in Italy published in 2023 showed that 28% of the patients still used betahistine as the main treatment (5). Further, it is the most frequently given drug for the treatment of vertigo according to a worldwide survey (6). Higher doses of up to 480 mg/d have shown benefit for severe cases in a small case series of Meniere's disease (7). This indicates that effectiveness seems to be a question of dosage and thereby serum concentration. For other indications, higher single dosages of betahistine were already studied, e.g., 200 mg per day in patients with attention deficit hyperactivity syndrome (8).

A basic pharmacokinetic problem of betahistine is that 99% of orally ingested betahistine is metabolized in the gastrointestinal tract and liver by MAO-B/A (9). This was also shown in a phase 1 trial in healthy volunteers (10). This first-pass effect can be reduced by MAO-B inhibitors, such as selegiline (11). Combination therapy like this is similar to the well-established combination of L-DOPA with carbidopa or benserazide in the treatment of Parkinson's disease.

In an animal model of unilateral vestibular neurectomized cats, oral treatment with betahistine in combination with selegiline led to significantly higher blood concentrations of betahistine and to a better posture function (12, 13). A study in guinea pigs demonstrated that cochlear microcirculation increased significantly after intravenous treatment with low-dose betahistine combined with selegiline compared to selegiline alone (14). An alternative approach to bypass the first-pass effect are intranasal (EudraCT 2018-002474-52) and transbuccal applications.

Based on these findings, the primary objective of this phase 1 trial in healthy subjects was to demonstrate that the serum concentration of betahistine is higher in a combination treatment with selegiline compared to betahistine monotherapy. The primary endpoint was the area under the curve (AUC0-240 min) of the serum concentrations of betahistine after combination treatment with betahistine and 5 mg/d selegiline compared to betahistine monotherapy, giving single dosages of 24, 48, and 96 mg betahistine in ascending dosage. The secondary objectives were the assessment of safety of the combination treatment and the pharmacokinetics of betahistine in different dosages in serum, namely the half-life time and the pharmacokinetic simulation of multiple dosing.

## 2. Methods

### 2.1. Study design

This study was an investigator-initiated (IIT), prospective, non-randomized, single sequence, two-period titration, open label single-center phase 1 study in healthy volunteers. It was conducted at the Department of Neurology, LMU University Hospital, LMU Munich, Munich, Germany with recruitment between 2 June 2021 and 9 September 2021.

### 2.2. Ethics approval and consent to participate

The study was performed in compliance with the requirements of the “Bundesinstitut für Arzneimittel und Medizinprodukte” (BfArM), Fachregistratur Klinische Pruefung, Kurt-Georg-Kiesinger-Allee 3, 53175 Bonn and Regierung von Oberbayern, Sachgebiet 55.2, 80534 Munich. It gained full regulatory approval from the Bundesinstitut für Arzneimittel und Medizinprodukte on 05.06.2020. The study was approved by the local ethics committees in Munich (19-992) on 25.05.2020. In the EudraCT register, the study was issued with the EudraCT number 2019-002610-39, registered on 17 June 2019 (first patient in 2 June 2021), named PK-BeST. As phase 1 studies are not public in the EudraCT register, the study was also registered in ClinicalTrials.gov.

### 2.3. Participants

Subjects were recruited via an announcement on the clinic homepage/intranet and were adult (age ≥18 years and ≤ 70 years) healthy volunteers (males and non-pregnant, non-breastfeeding women with adequate contraception) as determined by screening and the principal investigator's judgement. Participants were not allowed to take medication due to an illness and had normal blood pressure, heart rate, and ECG values.

Exclusion criteria were conditions in which repeated serum draws or injections pose more than minimal risk for the subjects, such as hemophilia, a positive test for alcohol or drugs, known contraindications for betahistine, such as bronchial asthma, and known contraindications for selegiline, e.g., hypersensitivity.

Each subject gave their written informed consent before any study-related procedures were performed.

### 2.4. Procedures

Study visits took place in an outpatient setting. The first step was to check the inclusion criteria at screening.

Subjects received single dosages of betahistine orally (24 mg, 48 mg, and 96 mg) with an at least 48-h wash-out period between the different dosages. In the second period, subjects were pre-treated with selegiline orally (5 mg/day) for 1 week and treated continuously with selegiline (5 mg/day) in combination with the single dosages of betahistine (24 mg, 48 mg, and 96 mg) with at least 2 days between the different dosages.

After absorption, betahistine is rapidly and almost completely metabolized into 2-PAA (which has no pharmacological activity in humans). After oral administration of betahistine, the plasma (and urinary) concentration of 2-PAA reaches its maximum 1 h after intake and declines with a half-life of about 3.5 h. A wash-out-time of 48 h, i.e., 15 times the half-life time, is sufficient and was feasible.

Vital signs and adverse events were checked for a minimum of 4 h after the intake of betahistine in the center. Serum samples to test serum concentrations of betahistine were obtained before and 10, 30, 60, 90, 120, 180, and 240 min after betahistine intake.

Safety parameters included assessment of adverse events, ECG, vital signs, laboratory measurements including kidney and liver function, full blood count, and pregnancy and drug screening test. Safety monitoring was conducted during every visit and assessment of vital parameters and adverse events was conducted 10, 30, 60, 90, 120, 180, and 240 min after betahistine intake. The clinical trial was conducted in a hospital of maximum care with the possibility to consult a specialist for possible symptoms of intoxication, specifically an anesthesiologist, cardiologist, and neurologist 24 h/7 days a week.

### 2.5. Randomization and masking

This was a phase 1 open label study. All subjects received the same treatment and no blinding process was involved. Due to safety reasons, ascending dosages were required by the federal institute so that no randomization was possible.

### 2.6. Outcomes

The primary endpoint was the area under the curve (AUC0-240 min) of the serum concentrations of betahistine after combination treatment with betahistine and 5 mg/d selegiline compared to betahistine monotherapy, giving single dosages of betahistine of 24, 48, and 96 mg in ascending dosage. Secondary endpoints were the half-life of betahistine in serum after intake of different dosages of betahistine and the occurrence of adverse effects due to combination treatment with betahistine and selegiline.

### 2.7. Simulations of multiple dosing of the three dosages examined

Computer simulations of multiple dosing with the three different dosages of betahistine and dosing regimens (three times per day and four times per day) were performed *post-hoc* to evaluate a possible accumulation over time. The simulations of multiple dosing regimens were conducted using a one-compartment model with PKTool, a free pharmacokinetic prediction tool developed with funding from the Bill & Melinda Gates Foundation (^*^https://www.mmv.org/research-development/computationalchemistry). As certain PK parameters necessary for simulating multiple dosing are unknown from empirical studies (e.g., Vd and F), estimates were obtained by fitting values to a one-compartment model using the single dose data with PKsolver (14).

### 2.8. Determination of sample size

The sample size chosen for this study was not based on statistical considerations. The number of subjects per dosage was chosen based on historical experience with safety and tolerance trials. The sample size falls within the range of those used in other studies of this nature. It was planned to enroll a total number of 15 subjects between 18 and 70 years of age in this trial at one trial site.

### 2.9. Statistical analysis

#### 2.9.1. Preliminary analysis

Our preliminary analysis includes descriptive statistics of demographics, general physical examination, relevant medical history, and concomitant medication and neurological examinations. Numerical variables (e.g., age, BMI, serum pressure, and heart rate) are summarized in terms of mean (SD), median (minimum-maximum), and median [1st Qu., 3rd Qu.]. Categorical variables (e.g., gender and neurological abnormalities) are summarized in terms of frequency counts and percentages.

#### 2.9.2. Preparatory graphical analysis

For our study, extensive graphical analyses preceded every population model analysis. Concentration-time data was plotted by individuals (one plot for each individual). Plots of the summarized data are also presented.

For pharmacokinetic parameters (AUC, half-life, elimination rate, *T*max*ax*, and *C*max*ax*), summary statistics are presented in a table followed by scatter plots on a linear y-axis and a log y-axis and bar charts. This is followed by the normality tests for linear and log transformed data.

#### 2.9.3. Analysis of primary outcome

This trial aims to demonstrate that serum concentration of betahistine is higher due to combination treatment with selegiline compared to betahistine. The superiority of the combination treatment with selegiline compared to betahistine alone is based on the area under the curve (AUC0-240 min) of the serum concentrations of betahistine after combination treatment with betahistine and 5 mg/d selegiline compared to betahistine monotherapy, giving single dosages of betahistine of 24, 48, and 96 mg in ascending order. The efficacy of the experimental intervention was determined by AUC0-240 min of betahistine before and 10, 30, 60, 90, 120, 180, and 240 min after treatment in the two treatment arms.

All data were plotted as grouped (average of all participants) and as individual data. The graphs of each individual's serum betahistine concentration over time was used to detect possible errant data points. The data from individual plots were then used to calculate the pharmacokinetic parameters (AUC, half-life, elimination rate, *T*max*ax*, and *C*max*ax*). The values from each individual were then used to calculate a mean and standard error of the mean.

The aforementioned pharmacokinetic parameters were plotted as individual points on scatter plots to see if the data was normally distributed and to evaluate the effect of treatment (dose ± selegiline).

For statistical analysis, as all pharmacokinetic parameters seemed to be not normally distributed, the data were log transformed. There was no evidence that the data were not log normal. All statistical tests were performed on the *L*og10 data.

Analysis of the primary endpoint was performed using repeated measures ANOVA on the *L*og10 transformed AUC0-240 min values. A repeated measure ANOVA was used with each participant used as a blocking factor (its own control to reduce signal to noise). To determine differences between means, rather than comparing all possible means, pre-planned comparisons (“*post hoc* tests”) were performed to determine the effect of selegiline on pharmacokinetics.

### 2.10. Role of the funding source

This study was funded by Cures within Reach (CwR), Chicago, USA. This trial was not industry co-sponsored. The funder of the trial had no role in the development of the study design, data collection, data analyses, data interpretation, or the writing of this report. All authors had full access to all data in the study and all authors had final responsibility for the decision to submit for publication.

## 3. Results

### 3.1. Participants

**Figure 1:**
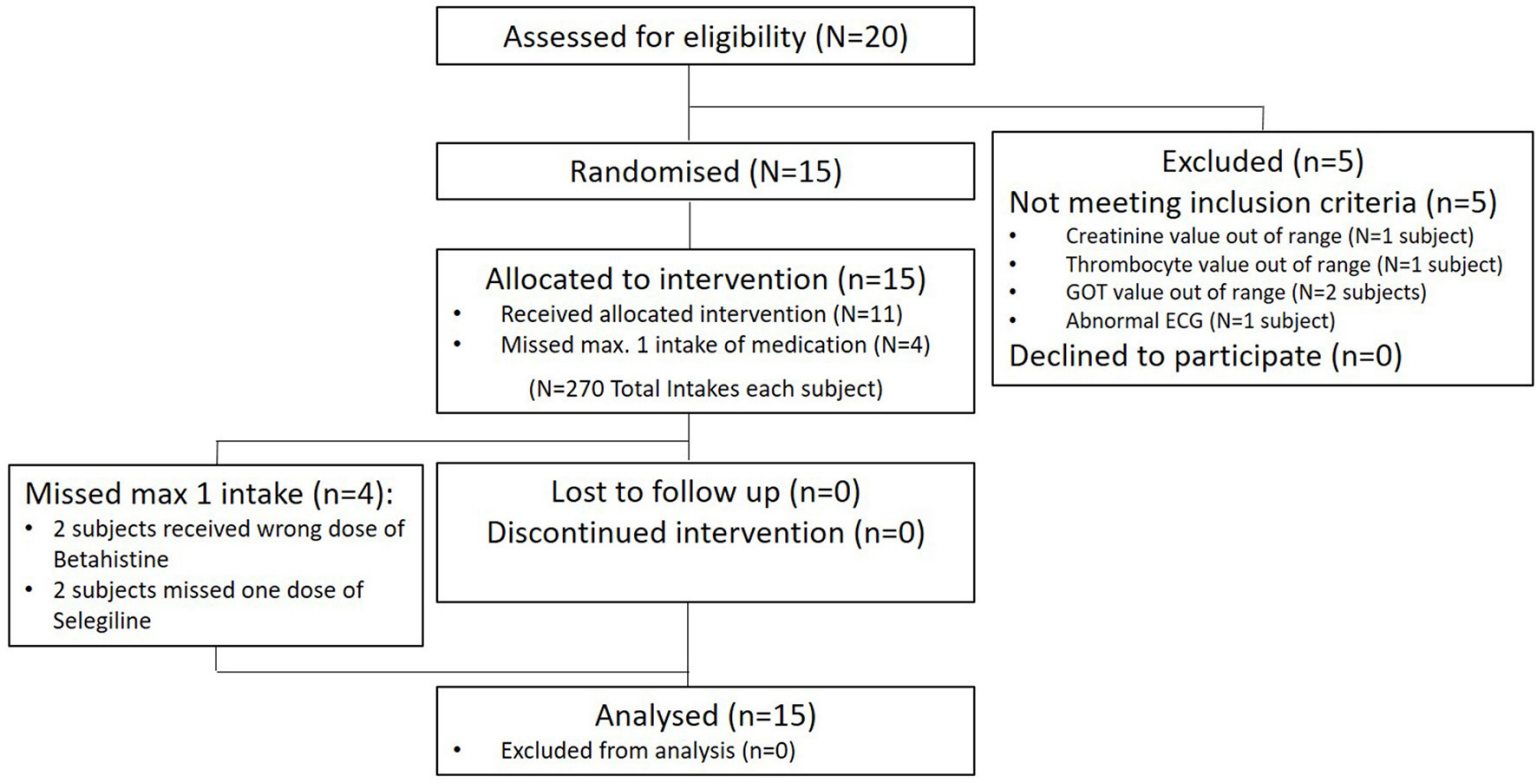
Participant flow.

Twenty healthy volunteers were screened for this study between June 2, 2021 and September 9, 2021. Five subjects failed to enter the study for different reasons, mostly laboratory findings or abnormal ECG values. Fifteen participants were enrolled. Of these, all completed the trial and were included in the analysis. For details, see Figure 1. There were no dropouts over the course of the whole trial.

### 3.2. Descriptive data

The baseline characteristics of the intention-to-treat sample include demographics, a general physical examination, relevant medical history, and concomitant medication and neurological examinations and are summarized in Table 1.

**Table 1:** Baseline characteristics of intention-to-treat sample.

**Characteristics**	**Subjects (*n* = 15)**
**Demographics**	
**Ethnicity**	
Caucasian	15.0 (100%)
**Gender [No. (%)]**	
Men	7.00 (46.7%)
Women	8.00 (53.3%)
**Age (years)**	
Mean (SD)	25.4 (6.37)
Median [Min, Max]	23.0 [20.0, 44.0]
Median [1st Qu., 3rd Qu.]	23.0 [22.0, 28.0]
**General physical examination**	
**Heart rate (pulse/min)**	
Mean (SD)	80.0 (9.88)
Median [Min, Max]	80.0 [61.0, 97.0]
Median [1st Qu., 3rd Qu.]	80.0 [73.0, 87.5]
**Systolic blood pressure (mmHg)**	
Mean (SD)	123 (12.1)
Median [Min, Max]	126 [96.0, 139]
Median [1st Qu., 3rd Qu.]	126 [117, 133]
**Diastolic blood pressure (mmHg)**	
Mean (SD)	79.3 (6.53)
Median [Min, Max]	80.0 [65.0, 88.0]
Median [1^st^ Qu., 3^rd^ Qu.]	80.0 [75.0, 85.0]
**Body Mass Index (kg/m** ^2^ **)**	
Mean (SD)	23.7 (4.29)
Median [Min, Max]	23.0 [18.4, 36.0]
Median [1^st^ Qu., 3^rd^ Qu.]	23.0 [20.8, 24.6]
**Relevant medical history [No (%)]**	
No	15.0 (100%)
**Use of concomitant medication [No (%)**	
No	15.0 (100%)
**Neurological examination**	
**Cranial nerve abnormalities [No (%)]**	
No	15.0 (100%)
**Motor nerve abnormalities [No (%)]**	
No	15.0 (100%)
**Sensitivity abnormalities [No (%)]**	
No	15.0 (100%)
**Coordination abnormalities [No (%)]**	
No	15.0 (100%)

### 3.3. Results of the primary endpoint

The mean AUC of betahistine seems to increase with the increased dose of administered betahistine and, more prominently, with the administration of a combination treatment of betahistine and selegiline.

**Figure 2:**
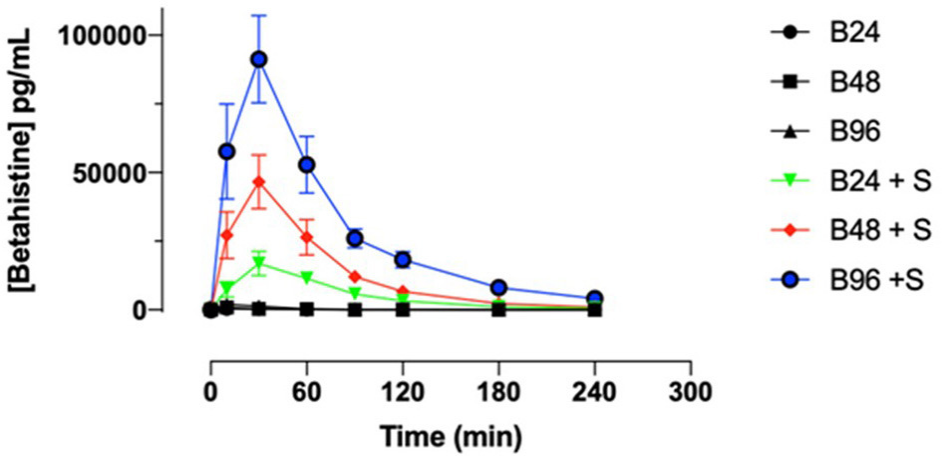
Selegiline increases the oral bioavailability of betahistine in humans as demonstrated by plots of serum concentrations of betahistine over time. Betahistine was orally dosed at 24, 48, and 96 mg/person (labeled on graph as B24, B48, and B96) alone or in combination with 5 mg selegiline (labeled on graph as B24 + S, B48 + S, and B96 + S). The total number of participants was 15 and each received all treatments.

Our analysis showed that the primary endpoint (mean AUC of betahistine) increased in all three betahistine dosages:

- 77-fold (95% CI: 42.3 to 141.5) by the combination of betahistine 24 mg plus selegiline 5 mg/d,
- 86-fold (95% CI: 52.9 to 138.9) by the combination of 48 mg betahistine plus selegiline 5 mg/d, and
- 108-fold (95% CI: 61 to 190.9) by the combination of 96 mg betahistine plus selegiline 5 mg/d compared to betahistine monotherapy (Figure 2).

For instance, the mean (+/-SD) of the area under curve for betahistine 48 mg alone was 0.64 (+/-0.47) h^*^ng/mL and for betahistine plus selegiline was 53.28 (+/-37.49) h^*^ng/ml. All the above results were statistically significant with a *P* < 0.0001, as shown in Table 2.

**Table 2:** Primary analysis for the ITT sample, mean *AUC*_0−240*min*_ of betahistine following the administration of different betahistine doses with and without selegiline using repeated one-way ANOVA on the *Log*_10_ data.

**Alpha**	**0.05**							
**Bonferroni's multiple comparisons test**	**Mean Diff**.	**95.00% CI of diff**.	**Statistically significant?**	**Adjusted** ***P*** **value**				
B24 vs. B24 + S	−1.889	−2.167 to −1.610	Yes	< 0.0001				
B48 vs. B48 + S	−1.934	−2.212 to −1.655	Yes	< 0.0001				
B96 vs. B96 + S	−2.034	−2.312 to −1.755	Yes	< 0.0001				
**Test details**	**Mean 1**	**Mean 2**	**Mean Diff**.	**SE of diff**.	**n1**	**n2**	**t**	**DF**
B24 vs. B24 + S	−0.6787	1.210	−1.889	0.1140	15	15	16.57	84
B48 vs. B48 + S	−0.2998	1.634	−1.934	0.1140	15	15	16.96	84
B96 vs. B96 + S	−0.02650	2.007	−2.034	0.1140	15	15	17.84	84

Analysis of the area under the curve of betahistine (0–240min after the intake of betahistine). Betahistine was orally dosed at 24, 48, and 96 mg/person (labeled as B24, B48, and B96) alone or in combination with 5 mg selegiline (labeled as +S).

**Figure 3:**
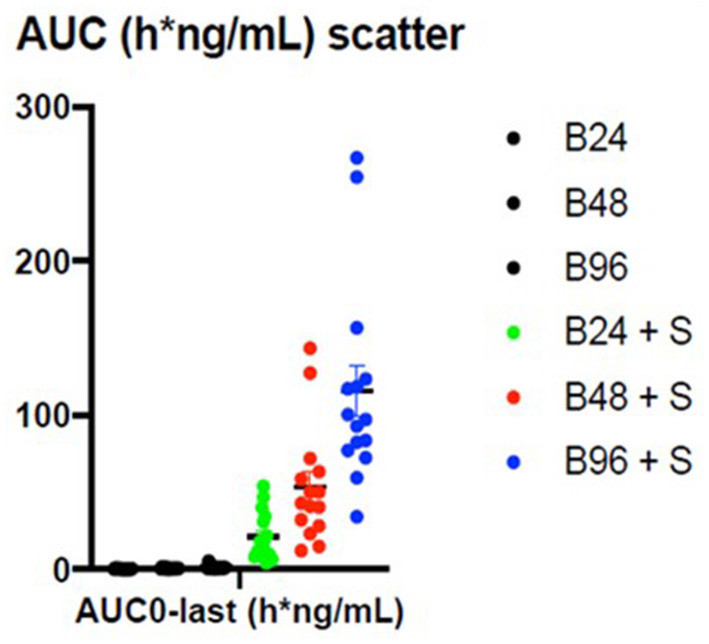
Scatter plots of the normal AUC_0−240_ min data in h*ng/ml following the administration of different betahistine doses with and without selegiline.

**Figure 4:**
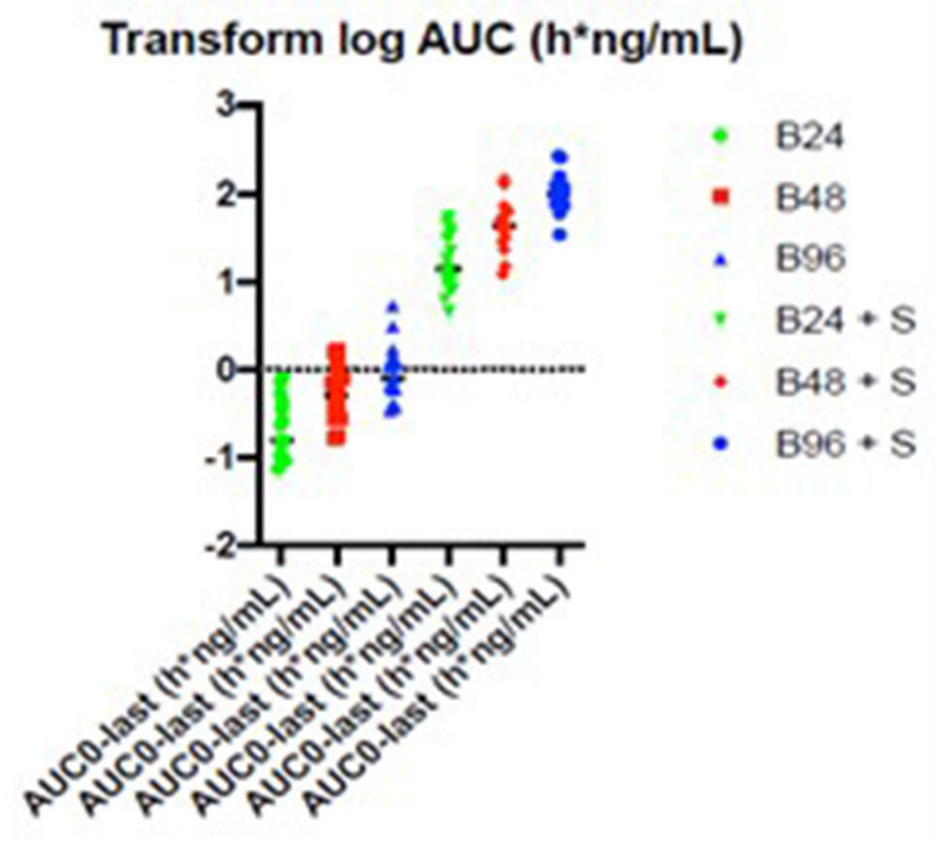
Scatter plots of the log-normal AUC_0−240_ min data in h*ng/ml following the administration of different betahistine doses with and without selegiline.

The summary statistics are shown in Table 3 and the details of the betahistine AUC0-240min in h^*^ng/ml are presented in Table 4. Figure 3 shows the scatter plots of the normal data and Figure 4 of log-normal AUC data following the administration of different betahistine doses with and without selegiline.

**Table 3:** Descriptive statistics.

**Descriptive statistics**		**B24**	**B48**	**B96**	**B24 + S**	**B48 + S**	**B96 + S**
**Number of values**		15	15	15	15	15	15
AUC (h^*^ng/ml)	Mean	0.2837	0.6406	1.293	21.31	53.28	115.7
	Std. deviation	0.2379	0.4696	1.312	15.97	37.49	65.61
Cmax (ng/ml)	Mean	0.5589	1.090	2.463	22.13	55.97	110.8
	Std. deviation	0.7435	1.254	2.634	14.20	35.40	68.16
Tmax	Mean	0.4556	0.7111	0.4667	0.6222	0.4778	0.5556
	Std. deviation	0.2707	0.7250	0.3684	0.3534	0.2587	0.2572
Thalf	Mean	0.2256	0.5757	0.3563	0.5041	0.5499	0.5081
	Std. deviation	0.2515	0.5595	0.2835	0.3832	0.4965	0.3640
ke	Mean	5.102	2.337	3.347	2.034	1.848	1.790
	Std. deviation	3.132	1.924	2.352	1.440	1.388	0.8376

Betahistine was orally dosed at 24, 48, and 96 mg/person (labeled as B24, B48 and B96) alone or in combination with 5 mg selegiline (labeled as +S).

AUC, Area under the curve of the betahistine serum concentration (0–240 min after intake of betahistine); Cmax, Maximum plasma concentration attained following dose administration; Tmax, Time at which the maximum concentration was reached; Thalf and ke, elimination rate. Mean, Arithmetic mean; SD, standard deviation.

**Table 4:** Descriptive Statistics of AUC_0−240*min*_ in h^*^ng/ml.

**AUC descriptive statistics**	**B24**	**B48**	**B96**	**B24 + S**	**B48 + S**	**B96 + S**
Number of values	15	15	15	15	15	15
Minimum	0.07360	0.1641	0.3441	4.482	12.06	34.12
25% percentile	0.1038	0.2684	0.5844	8.552	28.21	77.04
Median	0.1576	0.5115	0.8138	14.00	42.88	97.20
75% percentile	0.4240	0.7763	1.323	34.36	63.32	123.3
Maximum	0.8037	1.629	5.329	53.81	143.3	266.8
Range	0.7301	1.465	4.985	49.33	131.2	232.7
Mean	0.2837	0.6406	1.293	21.31	53.28	115.7
Std. deviation	0.2379	0.4696	1.312	15.97	37.49	65.61
Std. error of mean	0.06142	0.1213	0.3389	4.124	9.681	16.94
Lower 95% CI of mean	0.1520	0.3805	0.5662	12.46	32.52	79.37
Upper 95% CI of mean	0.4154	0.9006	2.020	30.15	74.04	152.0
Coefficient of variation	83.85%	73.31%	101.5%	74.96%	70.37%	56.70%
Skewness	1.251	1.158	2.466	0.8930	1.501	1.527
Kurtosis	0.4754	0.5014	6.525	−0.5052	1.932	1.907
Sum	4.255	9.609	19.40	319.6	799.2	1,736

Area under the curve of the betahistine serum concentration (0–240 min after intake of betahistine) in h^*^ng/ml. Betahistine was orally dosed at 24, 48, and 96 mg/person (labeled as B24, B48 and B96) alone or in combination with 5 mg selegiline (labeled as +S).

### 3.4. Results of the secondary endpoint

When examining the effect of the combination treatment of betahistine and selegiline vs. betahistine alone on the half-life of betahistine, a one-way repeated measure ANOVA and the post hoc tests (Bonferroni) showed a statistically significant increase in the half-life of betahistine following the combination treatment of betahistine 24 mg and selegiline 5 mg/d. The half-life of betahistine was 1.9 times higher, 95% CI (1 to 3.5) after a combination treatment of selegiline 5 mg/d with betahistine 24 mg. In contrast, there was no statistically significant difference in the half-life of betahistine between the combination treatment and the single treatment of betahistine 48 & 96 mg on the plasma half-life, as shown in Table 3 and the Supplementary Table S1.

When examining the effect of the combination treatment of selegiline and betahistine vs. betahistine alone on the Cmax of betahistine, a one-way repeated measure Anova and the *post hoc* tests (Bonferroni) showed a statistically significant 50-fold increase in Cmax of betahistine following the combination treatment of betahistine 24 mg and selegiline 5 mg/d.

There was no statistically significant difference between the combination treatment and the single treatment of betahistine 24, 48, and 96 mg on Tmax and elimination rate (ke) of betahistine.

### 3.5. Simulations of multiple dosing of the three dosages examined

**Figure 5:**
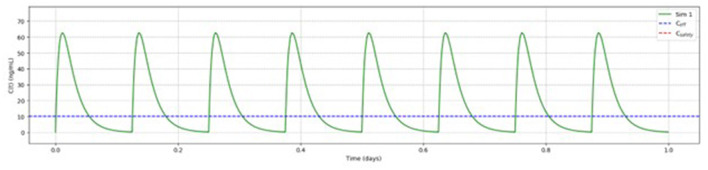
Pharmacokinetic modeling of 48 mg betahistine every 3 h (348 mg daily dose) and 5 mg selegiline once daily showed no accumulation of betahistine over time.

Based on the above reported findings, simulations of multiple doses with different dosages of betahistine/selegiline and dosing regiments were performed. Figure 5 shows as an example the results for betahistine 48 mg eight times per day (348 mg daily dose) and 5 mg selegiline once daily. This analysis demonstrated that there was no accumulation over time. The other simulations are shown in the Supplementary Figures S1–S3.

### 3.6. Safety results

In total, 14 adverse events (AE) were documented throughout the course of the trial (Table 5). None of them were assessed as serious, hence no SAE and SUSAR were observed. All AEs were assessed as mild, and the outcomes of all AEs were classified as recovered or resolved. No concomitant medication was taken by the subjects.

**Table 5:** Safety assessment and frequency of important adverse events.

**Safety assessment**	**Subjects (*n* = 15)**
No. of deaths	0
No. of subjects with SUSARs	0
No. of subjects with at least one SAE, total number of SAEs	0, 0%
No. of any AE	14
No. of Mild AEs	14 (100%)
No. of Moderate AEs	0, 0%
No. of Severe AEs	0, 0%
No. of recovered/resolved AE, total number of AEs	14 (100%), 14
No. of subjects with at least one AE, total number of study subjects	8 (53%), 15
No. of investigator-defined drug-related AE, total number of AEs	10 (71%), 14
Type of AE	
Headache	
No. of drug-related Headache AE, total number of Headache AEs	10 (83%), 12
Leukopenia	
No. of drug-related Leukopenia AE, total number of Leukopenia AEs	0 (0%), 1
Upper respiratory tract infection	
No. of drug-related upper respiratory tract infection AE, total number of upper respiratory tract infection AEs	0 (0%), 1

SUSARs, Suspected unexpected serious adverse reactions; SAE, Serious Adverse Event; AE, Adverse Event.

In total, 12 adverse events reported were classified as headache (nervous system; MedDRA Code 10019211) and of these, 10 AEs were assessed as related to the combination treatment of betahistine and selegiline. One adverse event was classified as an upper respiratory infection (infections and infestations; MedDRA Code 10046300) and assessed as not related to the trial treatment, and one adverse event was classified as leukopenia (blood and lymphatic system disorders; MedDRA Code 10024384) and assessed as not related to the trial treatment. All adverse events that were assessed as related to the treatment were headaches. These headaches started 15 min to a few hours after the intake of betahistine after pre-treatment with selegiline and lasted a few hours without intake of concomitant medication. No AEs related to the therapy were observed due to therapy with betahistine without selegiline and due to concomitant treatment with 5 mg selegiline and 24 mg betahistine. Headaches related to the treatment were observed in six out of 15 subjects due to concomitant treatment with 5 mg selegiline and 48 mg betahistine and in six out of 15 subjects upon treatment with 5 mg selegiline and 96 mg betahistine.

## 4. Discussion

The major findings of this phase 1 pharmacokinetic study in 15 healthy volunteers are:

Firstly, the MAO-B inhibitor selegiline (5 mg orally per day) increases the bioavailability of orally administered betahistine (in dosages of 24, 48 and 96 mg) by a factor of between 77 and 108.

Secondly, the half-life of around 30 min was largely unaffected; simulations showed that there was no accumulation over time with multiple doses.

Thirdly, 14 AE were documented throughout the course of the trial. All of these were assessed as mild, and the outcomes of all AEs were classified as recovered or resolved. Twelve adverse events reported were classified as headache and, of these, 10 AEs were assessed as related to the combination treatment of betahistine and selegiline.

Based on these findings and pharmacokinetic analysis, which showed an increase in the bioavailability of betahistine when combined with selegiline, as well as a favorable safety profile, phase II studies are needed and justified to evaluate the clinical efficacy. This combination can be useful for the preventive therapy of Menière's disease (7), acute treatment of acute unilateral vestibulopathy (12, 13, 15), and post-BPPV residual dizziness (16, 17), in the latter two to improve central compensation.

In particular, for the preventive treatment of Menière's disease there is a high unmet medical need because

a) of its high prevalence: it is the second most frequent peripheral vestibular disorder (the reported life-time prevalence of Menière's disease is between 34 and 190 per 100,000) (16–18).
b) Betahistine has already been used for decades in dosages of up to 48 mg tid, although it is not superior to placebo (2); this finding is supported by a Cochrane review (4). It is also not approved by the FDA.
c) There is insufficient evidence for the effectiveness of many other measures that have been recommended, such as life-style changes, e.g., low salt diet (19), diuretics (4), pulsed low-pressure delivery (20), surgical interventions (21), intratympanic steroids (22), and intratympanic gentamicin (23). All in all, there is very limited evidence for the efficacy of any prophylactic therapy in MD that does not impair vestibular function.

Finally, in terms of possible mechanisms of action of betahistine in Menière's disease, it was demonstrated in pre-clinical studies that it dose-dependently increases cochlear blood flow (24) which is mediated via the H3-receptor (25); selegiline alone did not have an impact on cochlear blood flow (14). In another animal model, it was found that betahistine can even prevent endolymphatic hydrops (26), which may have an impact clinically in terms of even preventing bilateral Menière's disease.

Combining our pharmacokinetic data with published pharmacodynamic data enables us to refine the proposed mechanism of action of betahistine. Specifically, we can compare the Cmax values with the published affinities of the histamine receptors to evaluate target engagement. Based on radioactive binding experiments, H1, H3, and H2 receptors had Kd values for betahistine of 64 μM, 2 μM, and millimolar, respectively (27). In other words, betahistine had an affinity to H1 and H3 but not H2 and H4 receptors. Using the Hill equation to calculate occupancy and using Cmax (from data with selegiline) as the concentration of betahistine, it can be seen that H1 receptors are only occupied between 0.2 and 1%, whereas at the same Cmax values the H3 receptors are occupied between 6 and 26% (Table 6). In other words, the H3 receptor is occupied 25 times more than H1, and H1 is only 1% occupied at the highest dose of betahistine in the presence of selegiline. We conclude that betahistine is acting through pre-synaptic H3 receptors (autoreceptors), which when blocked release more histamine (and other neurotransmitters) and also increase histamine synthesis (28), which could then act on H1 receptors. In terms of Menière's disease, such indirect activation of the H1 receptor, which is found in a high density in the human endolymphatic membrane (29), could lead to an increased membrane permeability and reduction of endolymphatic pressure. However, it must be said that the theories regarding the etiology of Menière's disease are still not finally clarified and therefore it remains disputable what the effects of betahistine will be with regards to the course of the disease. In other vestibular diseases, namely central compensation of acute unilateral vestibulopathy and central compensation of post-BPPV residual dizziness, its benefit could be via the (indirect) activation of H1 receptor, as was demonstrated in an animal model (13, 30, 31).

**Table 6:** Histamine receptor subtype occupancy during treatment with betahistine alone and betahistine combined with selegiline.

	**Betahistine**			**Betahistine** + **Selegiline**		
	**24 mg**	**48 mg**	**96 mg**	**24 mg**	**48 mg**	**96 mg**
Cmax CI_95_ (ng/mL)	0.22–0.58	0.38–1.19	0.89–2.68	13–26	32–67	67–130
Cmax mean (ng/mL)	0.36	0.67	1.54	18	46	94
Cmax mean (nM)	3	5	11	134	337	689
H1 occupancy (%)	0.0047	0.008	0.017	0.21	0.68	1.1
H3 occupancy (%)	0.13	0.25	0.56	6	14	26

Mean, geometric mean.

Finally, more broadly, these findings may also have an impact on the treatment of other diseases with betahistine. For instance, in high dosages it can improve cognition in schizophrenia (32).

### 4.1. Limitations

In the phase 1, single-center, open label study, only single dosages of betahistine and not the effects of repeated dosing was evaluated. However, the latter was studied by simulations. Only one dosage of selegiline was tested, which was half of the maximal dosage. As local authorities insist on ascending dosages for safety reasons, the study could not be randomized. For safety reasons, the sample size included only a small number of 15 healthy individuals.

Future phase 2 trials need to pay attention to contraindication of selegiline, a MAO-inhibitor, which can lead to serotonergic effects and cannot be combined with tricyclics (e.g., nortriptyline or amitriptyline) or SSRI/SNRI anti-anxiety antidepressants. This could limit the usability in patients with MD and migraine as well as depression. Both medications are well tolerated, and the side effect profile is well-defined. Side effects of betahistine include headache and gastric complaints; selegiline can lead to dizziness, headache, bradycardia, and a mild elevation of liver enzymes, so monitoring is necessary.

With regards to previous studies and analyses, which showed no effect of betahistine in MD in a dosage of up to 48 mg tid in comparison to placebo, and the uncertainties regarding the pathophysiology of Menière's disease and possible mode of action of betahistine in different diseases, it is uncertain if an increased bioavailability leads to a clinical effect. Therefore, placebo-controlled trials with a combination therapy in Menière's disease are needed. The same is true for acute vestibular syndrome or post-BPPV residual dizziness.

### 4.2. Conclusion

This phase 1 trial shows that the MAO-B inhibitor selegiline increases betahistine bioavailability with no safety concerns. Whether the increased bioavailability has an impact on the preventive treatment of Menière's disease, acute vestibular syndrome, or residual dizziness after BPPV has to be evaluated in placebo-controlled trials.

## Data availability statement

The original contributions presented in the study are included in the article/Supplementary material, further inquiries can be directed to the corresponding author.

## Ethics statement

The studies involving humans were approved by Local Ethics Committees in Munich (19-992). The studies were conducted in accordance with the local legislation and institutional requirements. The participants provided their written informed consent to participate in this study.

## Author contributions

MS: Conceptualization, Data curation, Funding acquisition, Investigation, Methodology, Project administration, Resources, Supervision, Writing—original draft. GC: Data curation, Formal analysis, Methodology, Validation, Visualization, Writing—original draft. IN: Investigation, Project administration, Resources, Writing—review and editing. UM: Supervision, Writing—review and editing. AA: Data curation, Formal analysis, Validation, Writing—review and editing. AG: Investigation, Writing—review and editing. NG: Investigation, Methodology, Project administration, Validation, Visualization, Writing—original draft.
