# Supplementary material for: Examination of betahistine bioavailability in combination with the monoamine oxidase B inhibitor, selegiline, in humans—a non-randomized, single-sequence, two-period titration, open label single-center phase 1 study (PK-BeST)
Source: Front Neurol. 2023 Oct 18;14:1271640. doi: 10.3389/fneur.2023.1271640 (PMC10619746; doi:10.3389/fneur.2023.1271640)
Supplement: Supplementary file 1 [file Data_Sheet_1.PDF]

Supplementary Material

Examination of betahistine bioavailability in combination with the monoamine oxidase-B inhibitor selegiline in humans – a non-randomised single sequence two period titration open label single centre phase 1 study (PK-BeST)

Michael Strupp[1], Grant C. Churchill[2], Ivonne Naumann[1], Ulrich Mansmann[3], Amani Altawil[3], Anastasia Golentsova[1], Nicolina Goldschagg[1]

Department of Neurology and German Center for Vertigo and Balance Disorders, LMU University Hospital, LMU Munich, Munich, Germany

[2]Dept of Pharmacology, University of Oxford, UK

[3]Dept. of Medical Information Sciences, Biometry and Epidemiology, Ludwig Maximilians University, Munich, Germany

*** Correspondence:**
Corresponding Author
Michael Strupp, MD, FRCP, FANA, FEAN, FAAN
Professor of Neurology and Clinical Neurophysiology
Department of Neurology
LMU University Hospital, LMU Munich
Marchioninistr. 15, 81377 Munich, Germany
Telephone: +49-89-4400-73678, Fax: +49-89-4400-76673
E-mail: Michael.Strupp@med.uni-muenchen.de

Table S1:

Secondary efficacy analysis for the ITT sample, mean Half Life of Betahistine following the administration of different Betahistine doses with and without Selegiline using Repeated One-way ANOVA on the Log10 Data

Alpha	0.05							
**Sidak's multiple comparisons test**	**Mean Diff.**	**95.00% CI of diff.**	**Significant?**	**Adjusted P Value**				
B24 vs. B24 + S	-0.2785	-0.5503 to -0.006781	Yes	0.0440				
B48 vs. B48 + S	0.02582	-0.6041 to 0.6557	No	0.9993				
B96 vs. B96 + S	-0.1518	-0.5113 to 0.2076	No	0.6085				
**Test details**	**Mean 1**	**Mean 2**	**Mean Diff.**	**SE of diff.**	**n1**	**n2**	**t**	**DF**
B24 vs. B24 + S	0.2256	0.5041	-0.2785	0.09928	15	14	2.805	13
B48 vs. B48 + S	0.5757	0.5499	0.02582	0.2241	13	14	0.1152	11
B96 vs. B96 + S	0.3563	0.5081	-0.1518	0.1313	15	14	1.156	13

Figure S1:

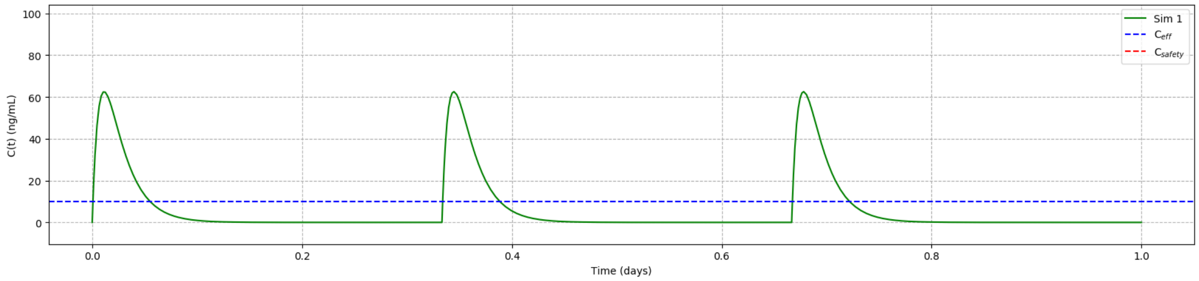

Figure S1:Pharmacokinetic modelling of 48 mg betahistine every 8 hours (144 mg daily dose) and 5 mg selegiline once daily showed no accumulation of betahistine over time.

Figure S2:

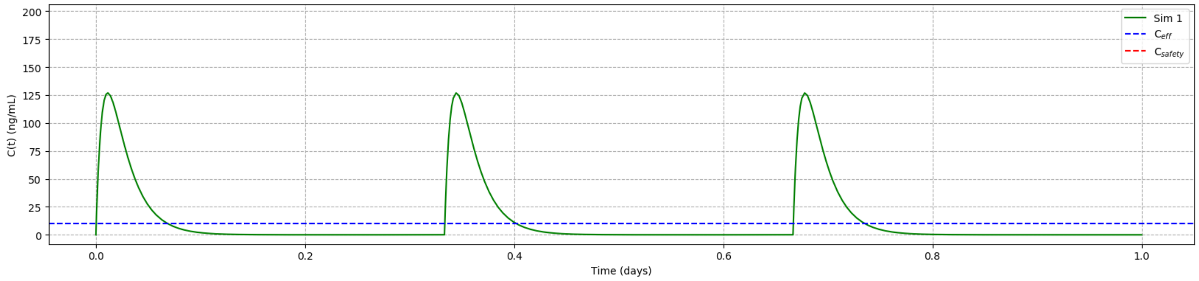

Figure S2: Pharmacokinetic modelling of 96 mg betahistine every 8 hours (288 mg daily dose) and 5 mg selegiline once daily showed no accumulation of betahistine over time.

Figure S3:

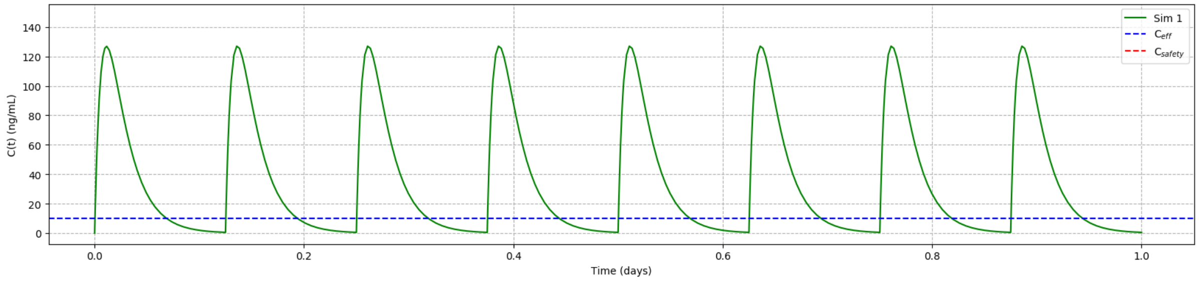

Figure S3: Pharmacokinetic modelling of 96 mg betahistine every 3 hours (768 mg daily dose) and 5 mg selegiline once daily showed no accumulation of betahistine over time.
